# Violent offences of methamphetamine users and dilemmas of forensic psychiatric assessment

**DOI:** 10.1080/20961790.2017.1287155

**Published:** 2017-02-16

**Authors:** Yi Liu, Bo Hao, Yanwei Shi, Li Xue, Xiaoguang Wang, Yefei Chen, Hu Zhao

**Affiliations:** a Faculty of Forensic Medicine, Zhongshan School of Medicine, Sun Yat-Sen University, Guangzhou, China; b Guangdong Province Key Laboratory of Brain Function and Disease, Zhongshan School of Medicine, Sun Yat-Sen University, Guangzhou, China; c Guangdong Province Translational Forensic Medicine Engineering Technology Research Center, Zhongshan School of Medicine, Sun Yat-Sen University, Guangzhou, China

**Keywords:** Forensic science, forensic psychiatry, methamphetamine, psychotic disorders, schizophrenia, violent offences

## Abstract

Methamphetamine (MA), an extremely addictive synthetic stimulant, has quickly spread to become the most frequently used illicit drug in China. People with a history of chronic and heavy MA use have a high possibility of exhibiting schizophrenia-like psychotic symptoms, mainly delusions of reference, auditory hallucinations and cognitive deficits. These emerging findings suggest MA use increases aggression and violence and that there is a correlation between MA use and violence. However, it is unclear how to assess the capacity of criminal responsibility in “MA-induced” psychosis and how to set clear boundaries between schizophrenia and MA-induced psychosis when only limited and inconsistent evidence is available. Furthermore, a final persuasive differential diagnostic method based on improved understanding of schizophrenia and MA-induced psychotic disorders has yet to be developed. This paper will evaluate the epidemiology, social harm, and forensic psychiatric assessment of MA users, propose a future direction for the differential diagnosis between MA-induced psychotic disorders and schizophrenia, and put forward some practical solutions to assess the capacity of criminal responsibility of defendants with drug-induced psychotic disorder.

## Introduction

More and more crimes related to mental disorders induced by synthetic stimulants, especially methamphetamine (MA), appear in forensic practice in recent years. Identification of criminal responsibility has been a controversial issue for offenders with drug-induced psychotic disorders. This review will try to propose a future direction for the differential diagnosis between MA-induced psychotic disorders and schizophrenia, and address the forensic implications of this work.

### Epidemiology of methamphetamine abuse

**Figure 1.**
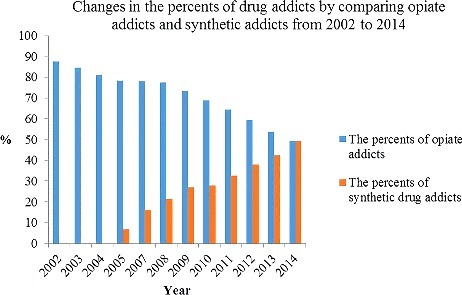
Percents of opiate addicts and synthetic drug addicts in drug addicts from 2002 to 2014 in mainland China. Data were from the State Council Information Office of the People's Republic of China (2014) [3] and China National Narcotics Control Commission [4].

According to official reports published by the United Nations Office on Drugs and Crime (UNODC), approximately 33.8 million people aged 15–64 years old used amphetamine-type stimulants in 2010 [1]. Amphetamine-type stimulants are the second-most commonly used illicit drugs worldwide [2]. East and South-East Asia are currently the most important consumer markets for these drugs and the number of MA users is steadily increasing in parts of North America and Europe [3]. A report from the State Council Information Office of the People's Republic of China (2014) suggested that over 14 million people have used an illicit drug in their lifetime in China [4]. Drug users younger than 35 years old account for 57.1% of all registered drug addicts. The number of MA users tends to increase year by year, and will soon possibly surpass the total number of opiate users. By the end of 2014, the total number of opiate addicts, including heroin, was 1.458 million, accounting for 49.3% of all registered drug addicts, whereas the total number of synthetic drug addicts was 49.4% at 1.459 million, within which MA addicts accounted for more than 80% (Figure 1) [4, 5]. It is the first time that the number of synthetic drug addicts is greater than that of opiate addicts, which indicates that a considerable change has occurred in the proportion of drugs abused in China.

### Social, psychiatric and behavioural harm arising from methamphetamine abuse

#### Social harm of methamphetamine abuse

The drug-abuse data from the national monitoring centre in 2014 revealed that the rate of HIV infection among synthetic drug abusers was 1.4%. Drug addicts' consumption costs an average of RMB 40 000–50 000 each year. The estimated actual number of drug users is over 10 million, which means there is RMB 500 billion lost each year in China to drug consumption [4]. MA abuse can lead to illegal behaviours [6] such as crime and violence, which causes increased incarcerations and other criminal-justice problems, like forensic psychiatric assessment [7]. With the rapid spread of synthetic drugs, extreme cases, such as suicide, self-harm, assault, drugged driving often occur because of drug-induced psychotic symptoms. About 149 000 criminal cases caused by drug addicts were uncovered in 2014, which accounted for 12.1% of all criminal cases during the same period, including 72 000 crimes of property tort, such as robbery and theft, and over 300 serious violent crimes including murder, kidnapping and rape [4].

The damage caused by MA use varies with dosage and frequency. When administrated at low to moderate doses (5–30 mg), MA can result in euphoria and hyperactivity [8]. However, growing evidence shows that at frequent and high doses MA can trigger psychotic episodes, including auditory and visual hallucinations, paranoid delusions and delusions of reference [9, 10]. Under the influence of such symptoms, MA users have higher risk of violent behaviour that may ultimately result in crime [11, 12]. Additionally, there is considerable evidence supporting the claim that MA use is closely related to several aspects of cognitive impairment [13, 14]. For example, deficit in inhibition causes increased risk-taking behaviour [14]. Thus, it is important to clarify whether MA use itself or psychotic symptoms and cognitive deficits induced by MA result in violent behaviour.

#### Schizophrenia-like psychotic symptoms

Researchers have been exploring whether amphetamines resulted in psychotic episodes since the 1950s [15]. Early experimental studies confirmed that intravenous administration of d-amphetamine could induce psychotic episodes [16]. The subsequent studies also showed psychotic episodes in healthy subjects or amphetamine abusers through different experiments [17-19]. A consistent conclusion is that a part of participants may develop schizophrenia-like symptoms, which mainly are delusions of reference. A review of amphetamine-type stimulant induced psychosis showed the rates of psychotic symptoms: paranoia (25%–75%), acousma (50%–80%), delusions of reference (15%–60%), schizophrenia first-rank symptoms (up to 50%), and negative symptoms (5%–30%) [20].

Although growing cases support that MA is closely associated with psychotic symptoms, direct evidence from prospective studies is still absent. In a prospective longitudinal study, 276 MA-dependent participants older than 16 years with psychotic symptoms were recruited to record the odds ratio (OR) of psychotic symptoms in three circumstances, thus revealing a dose-dependent effect between MA use and psychotic symptoms [21]. The OR of 1–15 days and 16–30 days of MA use versus one month of abstinence were 4.0 and 11.2, respectively [21]. Further related studies still need to be conducted.

#### Aggressive and violent behaviour in MA users

Violence and crimes caused by illicit drugs are an important public health concern, especially in China, which has a population of over 14 million drug addicts. Evidence has shown a correlation between MA use and violence [22,23]. Determining the mechanism for how MA contributes to violence would have a positive impact on prior intervention of social harmfulness, accurate diagnosis and appropriate treatment of MA psychosis.

A survey concerning MA-related emergency cases in the United States shows that MA users mostly complain of mental health issues (18.7%) and trauma (18.4%), and one-third of the cases require treatment with a sedative because of agitation and aggression [24]. Investigations of community samples of MA users also reveal high rates of aggression and hostility [25, 26]. A South African survey revealed that 87% of MA users experience interpersonal violence at least once [25]. In Australia, 82% of 400 regular MA and heroin users surveyed had committed violent crime in their lifetime, 41% in the first year of MA use [27]. Additionally, MA users have a higher incidence of violence than heroin users (OR is 1.94) [27]. This conclusion has prominent social significance and practical value for China, because opiates will be possibly surpassed by amphetamine-type stimulants as the country's most popular drug recently.

There are three main possible underlying mechanisms of violent behaviour induced by MA. First, the neurotoxic and pharmacological impact of MA on the serotonergic systems is associated with aggressive and violent behaviour. Second, the positive symptoms of MA-induced psychosis, especially paranoia, result in an individual misinterpreting a benign environment as one that is hostile and threatening. Third, MA use is related to impairment of cognitive function in the prefrontal cortex [12,22].

It is well-known that MA might lead to the release of serotonin and impairment of serotonin systems [28,29]. Animal studies demonstrate that a high dosage and chronic use of MA could result in serotonin depletion [30]. Using positron emission tomography (PET), Sekine et al. [31] found that MA abuse reduced the density of serotonin transporter in global brain regions, which presented a negative relationship. Furthermore, the density of serotonin transporter in an MA user decreases 30% compared with that of non-users, even after a long period of abstinence. In addition, there is a significant association between the degree of aggression and the density of serotonin transporter, which is consistent with other studies concerning aggressive psychiatric patients [32], violent men [33] and violent offenders [34].

Most MA users experience schizophrenia-like psychotic symptoms; however, whether MA itself or MA-induced psychotic symptoms cause violence is still unclear. Lapworth et al. [12] thought the positive symptoms induced by MA were highly relevant to hostility and impulsivity. Furthermore, the positive symptoms interacting with impulsivity could incur higher levels of aggression. Considering that so many MA users initially ascribed to MA psychosis are ultimately diagnosed with schizophrenia [35,36] and that the heterogeneity between MA psychosis and schizophrenia has been unclear, it is important to investigate the level of violence among MA users diagnosed with schizophrenia. A systemic review and meta-analysis revealed the risk of interpersonal violent behaviour and/or violent criminality in individuals with schizophrenia and other psychoses from 1970 to 2009, confirming that random-effects odds risk of individuals with comorbidity psychosis and substance abuse (i.e. 8.9) was much higher than that of those without comorbidity (i.e. 2.1) [37]. Interestingly, there was a similar level of violent risk in individuals suffering from substance abuse with and without psychosis [37], which implied that substance abuse played a major role in violence. However, this study did not differentiate the timeline between drug abuse and psychotic episodes, nor did it analyse the relationship between different types of drugs and violence. Another outstanding prospective study from McKetin et al. [23] demonstrated that the risk rate of violence increased from 10% to 60% in relation to various degrees of MA use ranging from abstinence to heavy use. Unexpectedly, compared with MA use, psychotic symptoms induced by MA only accounted for 22%–30% of violent behaviour, which suggested violent behaviour was mainly influenced by MA use [23].

There are an increasing number of studies concerning the association of MA use with deficits in several cognitive domains, including perception, attention, self-control and memory [13,38]. All substance use is associated with heightened impulsivity and disinhibition [39], which implies that behavioural disinhibition may strengthen the correlation between aggression and amphetamine-type stimulants [12]. During the performance on the Stop-Signal Task, MA dependent users gave slower responses of inhibition than control users [40]. MA nondependent users tended to yield to the “action pressure” while making decisions under uncertainty [41]. Remarkably, even if male individuals have been abstinent for an average of 19 months, executive function related to behaviour inhibition remains impaired [42], indicating that cognitive impairment caused by MA use lasted long beyond the duration of the pharmacological effect. Although these relevant studies could not directly ascertain impulsivity and disinhibition as consequences of MA use, it is unambiguous that impulsivity and disinhibition are predictors of aggressive behaviour. In addition, several predictive factors should be considered, such as the age of MA abusers at the time of their first violent act [43], family deviance [44], and psychosocial factors [45,46].

### Debate surrounding diagnosis of methamphetamine psychosis

As of now, researchers of different countries have yet to reach an agreement on the issue of how long MA-induced psychosis lasts. Early studies suggested that an individual could recover from MA psychosis in an average of one week [14–17]. However, there are increasingly inconsistent reported cases [47] and epidemiological surveys [10] challenging this conclusion. The *Diagnostic and Statistical Manual of Mental Disorders*, Fifth Edition (DSM-5) states that substance-induced mental disorders are likely to disappear within one month of abstinence [48]. Similarly, based upon the regulations in the *International Classification of Diseases*, Tenth Revision, if individuals discontinue drug use, psychotic symptoms due to psychoactive substance use mostly disappear in a month and do not last more than six months [49]. However, psychotic symptoms of individuals withdrawing from MA last up to one month or longer, and some users are eventually diagnosed with primary psychotic disorders such as schizophrenia [50]. A large epidemiological survey in Thailand showed that over 1 000 MA users had suffered psychotic disorders caused by MA, nearly 40% of which were diagnosed with schizophrenia due to persistent psychosis within 6 years after the first reported episode [35]. From a total of 18 478 recruited patients with drug-induced psychotic disorders treated from 1990 to 2000 in Finland, the 8-year cumulative risk of inpatients diagnosed with amphetamine psychosis being diagnosed with schizophrenia spectrum disorder was 30% [36]. In Japan, Ujike and Sato et al. [10] classified MA psychosis into two types: transient-type, which recovered within one week or no longer than one month, and prolonged and persistent type, in which an individual would return to normal after one month, occasionally after 6 months. Forty-one per cent of MA users exhibited persistent MA psychosis, approximately 28% of which experienced psychotic symptoms lasting longer than six months [10].

There are two mainstream theories of the underlying mechanism development of chronic and persistent psychosis in some MA users. The MA use could trigger a pre-existing schizophrenia as described in DSM-5, or persistent MA psychosis and schizophrenia are two separate diseases displaying a similar clinical course [51]. Combining the stress-vulnerability [52,53] with dopamine sensitization [54,55] hypotheses of schizophrenia, the third theory argues that all addictive drugs are “stressors” that affect the dopamine system and hence make people vulnerable to schizophrenia [56]. However, this theory cannot explain why MA use has a higher possibility of persistent psychosis than the use of cocaine, opioids, and alcohol [57]. Therefore, given the variety of duration and intricateness of the mechanisms underlying MA psychosis, the diagnostic criteria in DSM-5 need to be improved [58].

An improved persuasive differential diagnostic method depends on further understanding of schizophrenia and MA-induced psychotic disorders. To inform the diagnostic differentiation between MA-induced and independent psychotic symptoms, two kinds of information are necessary: (a) identification of early markers that clearly differentiate the two conditions and (b) more precise information about the duration of MA-induced psychotic symptoms. At present, the most definitive method for making this distinction is longitudinal assessment after a period of sustained abstinence from MA. It is also necessary to accurately assess the temporal link between substance use and the psychotic episode.

General reasons for difficulties in differential diagnosis include unsustainable drug-free periods, inconsistencies in substance users' reports, poor memory of the precise sequence of events and difficulty in cooperating with the examiner [58,59]. Related data should be collected from multiple sources to improve diagnostic accuracy [50]. This could include interviews with someone close to a substance user; a survey of medical records; outcomes of objective indicators such as urine toxicology tests; structured interview assessments with a substance user such as the Psychiatric Research Interview for Substance and Mental Disorders (PRISM) [60]; the diagnostic interview schedule (DIS) [61] and the composite international diagnostic interview (CIDI) [62]; and identification of some early markers (e.g. a genetic profile suggesting schizophrenia, among others) [58]. Considering the existing complications, the best way of distinguishing MA psychosis from other psychotic disorders is to analyse various data and then make a final diagnosis.

### Forensic psychiatric assessment of methamphetamine-induced psychotic disorders

In forensic psychiatry, the issue of appraising criminal responsibility among individuals with mental disorders induced by drugs is always challenging. The major focus of the debate is how to determine criminal responsibility when drug users displaying obvious psychotic symptoms commit dangerous acts in a state without cognitive and volitional capacity, similar to that of a “common mental patient.” MA abuse is character of the action liberal in cause, so the defendants undergoing prosecution for a criminal act should be regarded as having full capacity for criminal responsibility. However, as schizophrenia and bipolar disorder are endogenous mental diseases, the patients have no or partial capacity for criminal responsibility according to Chinese guidelines for the assessment of criminal responsibility of mentally ill patients.

Today, forensic assessment of capacity for criminal responsibility of “MA-induced” psychosis is one of the most controversial issues in the field. Most substance-induced psychotic symptoms are considered to be short-lived and to resolve with sustained abstinence along with other symptoms of substance intoxication and withdrawal. However, the largest problem facing this assessment is a defendant's remaining psychotic symptoms after withdrawal from MA. Even after periods of normality, a subpopulation of patients with MA psychosis showed spontaneous recurrence. In addition to the everyday practical challenges of differentiating “MA-induced” from “independent” psychotic disorders, a major issue related to the etiology of psychotic disorders is whether MA use can be considered a “cause” of schizophrenia, which has been traditionally thought of as “independent” of substance use. Thus, in many circumstances the question remains: are MA-induced psychotic disorders and schizophrenia the same disease, or two completely different diseases with similar clinical manifestation? For now, the generally accepted definition of MA psychosis is that of a broad spectrum of drug-associated psychiatric disorders regardless of length of abstinence. Currently, it is difficult to set clear boundaries between schizophrenia and MA psychosis owing to limited and inconsistent evidence. Based on current diagnostic criteria, approximately 30%–40% of patients who are first diagnosed with MA psychosis are ultimately diagnosed with primary psychotic disorders such as schizophrenia [35,36]. From the perspective of judicial practice, those individuals who experience persistent psychotic symptoms after long abstinence of MA (more than 6 months) should be regarded as “independent” psychotic disorders during appraisal of their capacity for criminal responsibility.

To facilitate accurate identification within forensic psychiatry, the feasible solutions to appraising criminal responsibility among individuals with MA-induced psychotic symptoms are set as follows: First, in accordance with the method described previously, detailed information regarding history of substance use and psychotic episodes should be collected. Then, a final medical diagnosis should be made from comprehensive analysis of the clinical manifestations of psychotic symptoms. If defendants still experience various psychotic symptoms more than six months after MA withdrawal, it is highly recommended for the defendant to be diagnosed with an “independent” psychotic disorder. Finally, criminal responsibility of the defendant should be evaluated from the perspective of substantial capacity to identify the criminality of his conduct and conform his conduct under the requirements of the law.

## Conclusion

In China, there is an urgent need to strengthen government administration to prevent the prevalence of MA. Compared with other addictive drugs (except for marijuana) [32], MA abuse has a greater possibility of schizophrenia-like psychotic symptoms that generally disappear within a month after use has stopped. However, some MA users experience persistent psychotic symptoms after more than 6 months. The etiology, pathogenesis and clinical course of MA psychosis are unclear, which increases the difficulty of personalized diagnosis, treatment and forensic psychiatric assessment. Remarkably, a variety of evidence from simple description, epidemiology, animal experiments and imaging researches reveal MA use can increase violent behaviour. Hence, MA use is highly associated with violent crime. Three main risk factors of violence related to MA are the magnitude of MA use, MA-induced psychotic symptoms and cognitive impairment. Heavy use of MA is usually accompanied by transient or persistent psychotic symptoms, which increases the difficulty of assessment of the cognitive and volitional capacity of a defendant as well as appraising competence for criminal responsibility. As MA problems have brought a heavy burden to families, greater society, and the judicial system, we should increase efforts to adequately address this challenge.
